# Scintillation and Optical Characterization of CsCu_2_I_3_ Single Crystals from 10 to 400 K

**DOI:** 10.1021/acs.chemmater.3c01810

**Published:** 2023-11-09

**Authors:** J. Jasper van Blaaderen, Liselotte A. van den Brekel, Karl W. Krämer, Pieter Dorenbos

**Affiliations:** † Faculty of Applied Sciences, Department of Radiation Science and Technology, Delft University of Technology, Mekelweg 15, 2629 JB Delft, Netherlands; ‡ Department of Chemistry, Biochemistry and Pharmacy, Univeristy of Bern, Freiestrasse 3, 3012 Bern, Switzerland

## Abstract

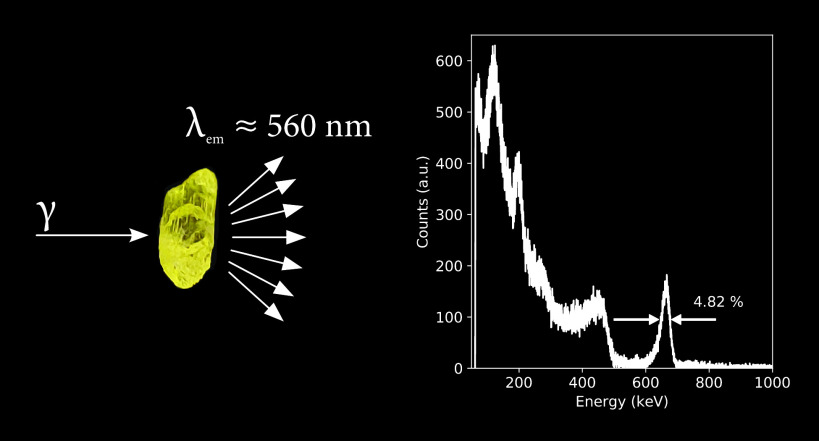

Currently only Eu^2+^-based scintillators have approached the light yield needed to improve the 2% energy resolution at 662 keV of LaBr_3_:Ce^3+^,Sr^2+^. Their major limitation, however, is the significant self-absorption due to Eu^2+^. CsCu_2_I_3_ is an interesting new small band gap scintillator. It is nonhygroscopic and nontoxic, melts congruently, and has an extremely low afterglow, a density of 5.01 g/cm^3^, and an effective atomic number of 50.6. It shows self-trapped exciton emission at room temperature. The large Stokes shift of this emission ensures that this material is not sensitive to self-absorption, tackling one of the major problems of Eu^2+^-based scintillators. An avalanche photo diode, whose optimal detection efficiency matches the 570 nm mean emission wavelength of CsCu_2_I_3_, was used to measure pulse height spectra. From the latter, a light yield of 36 000 photons/MeV and energy resolution of 4.82% were obtained. The scintillation proportionality of CsCu_2_I_3_ was found to be on par with that of SrI_2_:Eu^2+^. Based on temperature-dependent emission and decay measurements, it was demonstrated that CsCu_2_I_3_ emission is already about 50% quenched at room temperature. Using temperature-dependent pulse height measurements, it is shown that the light yield can be increased up to 60 000 photons/MeV by cooling to 200 K, experimentally demonstrating the scintillation potential of CsCu_2_I_3_. Below this temperature, the light yield starts to decrease, which can be linked to the unusually large increase in the band gap energy of CsCu_2_I_3_.

## 1 Introduction

Scintillation research in the past 30 years has mainly focused on the development of Ce^3+^- and Eu^2+^-doped materials.^1^ The energy resolution record of 2% at 662 keV γ-energy, achieved by Alekhin et al. in 2013 using LaBr_3_:Ce^3+^,Sr^2+^,^2^ still stands today. This resolution approaches the fundamental energy resolution limit determined by photon statistics. It could be surpassed by either increasing the number of photons detected in a scintillation event or increasing the light yield beyond the 70 000 photons/MeV reported for LaBr_3_:Ce^3+^,Sr^2+^.^2^

There are several Eu^2+^-doped halide scintillators that have surpassed the light yield of LaBr_3_:Ce^3+^,Sr^2+^. Examples are CsBa_2_I_5_:Eu^2+^^3−6^ and SrI_2_:Eu^2+^^7−11^ with reported light yields of 100 000 and 115 000 photons/MeV and energy resolutions of 2.6% and 2.3%, respectively. Despite these very promising numbers, Eu^2+^-based scintillators suffer from two major drawbacks: self-absorption and concentration quenching.^4,12−16^

These problems can be mitigated by using a codoping strategy based on Sm^2+^,^17−19^ transferring almost all excitations from Eu^2+^ to Sm^2+^. This produces only Sm^2+^ emission and limits self-absorption losses. Additionally, this shifts the mean emission wavelength to longer wavelengths, around 700 to 850 nm, allowing the use of modern Si-based photodetectors.^17^ The latter have higher detection efficiencies compared to more traditional photomultiplier tubes, enabling them to detect more photons from a scintillation event. This wavelength shifting effect has also been demonstrated for Yb^2+^ to Sm^2+^.^20,21^

More recently, intrinsic small band gap materials have gained significant traction in scintillation research. Hybrid organic–inorganic perovskites (HOIP) are a good example of such a group of materials.^22−26^ The small band gap of these materials significantly increases their theoretical scintillation light yield compared to more traditional scintillators.^1,27,28^ In particular, intrinsic small band gap materials showing self-trapped exciton (STE) emission are very promising candidates. The strong electron–phonon coupling in these materials creates a large Stokes shift resulting in self-absorption-free emission, solving the problem of Eu^2+^-based scintillators. Examples of such compounds are Rb_2_CuCl_3_.^29^ Rb_2_CuBr_3_,^30^ and Cs_3_Cu_2_I_5_.^31−33^ The latter has shown especially promising scintillation properties, with an energy resolution of 3.4% and a light yield of 29.000 photons/MeV.^31^

In this work the emerging intrinsic small band gap scintillator CsCu_2_I_3_ is characterized as a function of temperature. Currently this material has mainly been studied under UV–vis excitation at room temperature for optoelectronic applications, with some scintillation-related studies appearing in recent years.^34−39^ Cheng et al. have performed a room-temperature scintillation characterization of this material, showing an energy resolution of 7.8%, a light yield of 16 000 photons/MeV measured on a photomultiplier tube (PMT), and low afterglow level of 0.008% at 10 ms.^40^ Liu et al. and Shu et al. have explored the influence of doping CsCu_2_I_3_ with Li^+^ and Na^+^, respectively, only finding minor improvements of the quantum yield at room temperature.^41,42^ Zhang et al. have explored the use of CsCu_2_I_3_ for imaging applications.^43^

CsCu_2_I_3_ has many advantageous scintillator properties,; it has a density of 5.01 g/cm^3^ and *Z*_eff_ of 50.6. It melts congruently at 656 K,^44^ and is nonhygroscopic and nontoxic.^36,40^ Although the quantum yield of Cs_3_Cu_2_I_5_ is higher at room temperature, CsCu_2_I_3_ is chosen due to the better match of its mean emission wavelength with modern Si-based photodetectors.^17^ Additionally, Cs_3_Cu_2_I_5_ melts incongruently, complicating the growth of single crystals.^44^ The goal of this work is to study the scintillation and optical properties of CsCu_2_I_3_ from 400 to 10 K in order to develop a better understanding of the scintillation and photophysical properties of CsCu_2_I_3_.

## 2 Results

**Figure 1:**
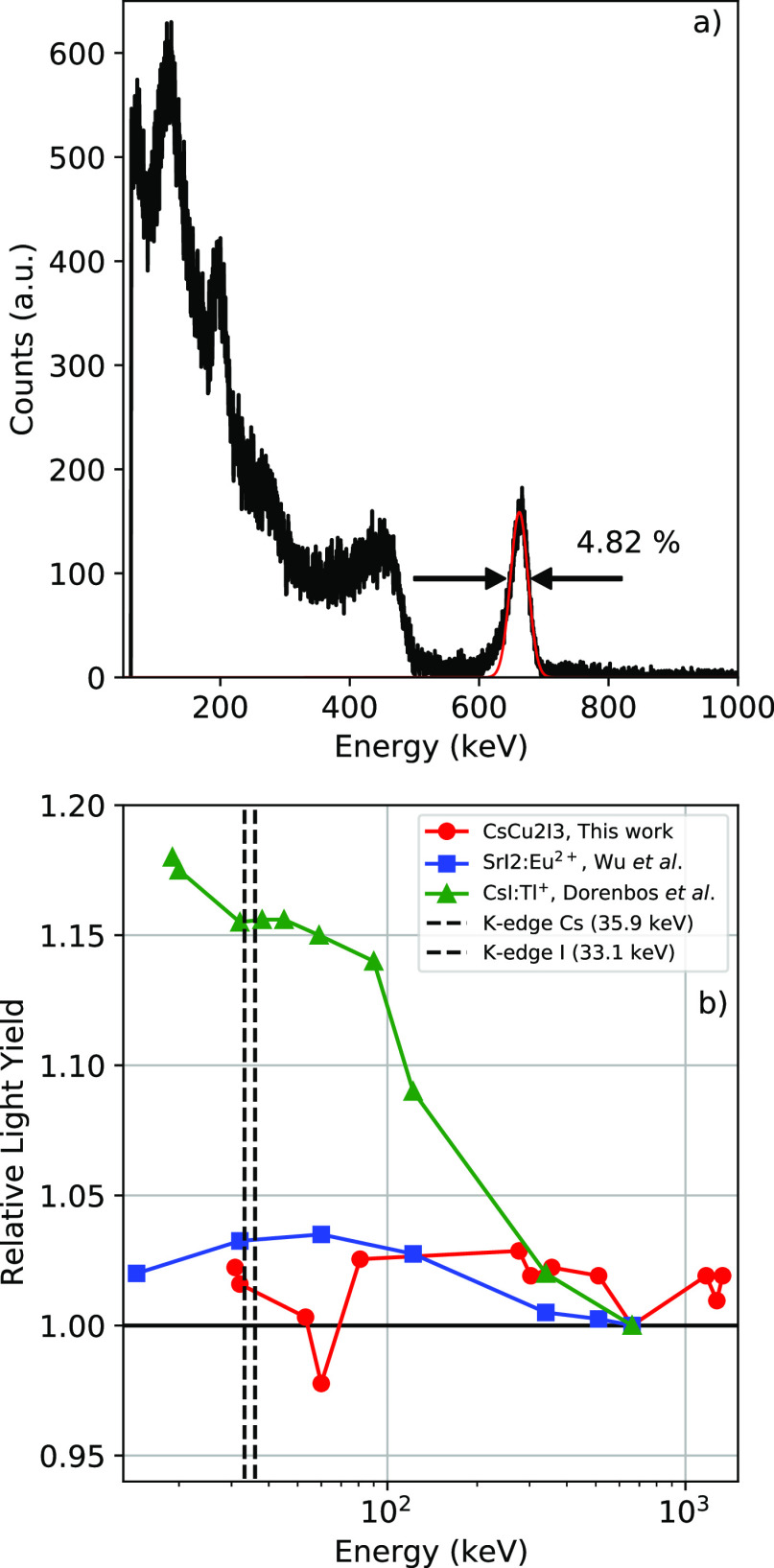
(a) Pulse height spectrum of a CsCu_2_I_3_ single crystal (10 mm × 3 mm × 3 mm) measured on an avalanche photo diode (APD) using a ^137^Cs γ-source. The red line in the plot shows a fitted Gaussian function used to obtain the energy resolution and light yield. (b) Nonproportional response of CsCu_2_I_3_ in comparison to those of SrI_2_:Eu^2+^^10^ and CsI:Tl^+^.^46^ The pulse height spectra were recorded using ^137^Cs, ^22^Na, ^133^Ba, ^60^Co, and ^241^Am. The ideal response is indicated by the horizontal line, at a relative light yield of 1. The K edges of Cs and I at 35.9 and 33.1 keV, respectively, are indicated by the vertical dashed lines.

Figure 1a shows the pulse height spectrum of a CsCu_2_I_3_ single crystal (10 mm × 3 mm × 3 mm) measured on an avalanche photo diode (APD) using the 662 keV γ-photons of ^137^Cs. An APD was used to match the detection efficiency to the mean emission wavelength of CsCu_2_I_3_, using the same approach as described by Wolszczak et al.^17^ Based on the full width at half-maximum (fwhm) of the total absorption peak, the energy resolution is determined to be 4.8%. The total absorption peak corresponds to the detection of 24 300 electron–hole pairs, based on which the light yield was determined to be 36 000 photons/MeV using the method described by De Haas and Dorenbos.^45^ This is a significant improvement compared to the values reported by Cheng et al., who performed their measurements on a PMT.^40^

The same CsCu_2_I_3_ sample was used to study the light yield as a function of deposition energy, employing γ-photons from ^137^Cs, ^22^Na, ^133^Ba, ^60^Co, and ^241^Am. The resulting proportionality curve is shown in Figure 1b. The proportionality curves of SrI_2_:Eu^2+^^10^ and CsI:Tl^+^^46^ are plotted as reference. An ideal response would be a straight horizontal line at a relative light yield of 1, as indicated by the black horizontal line in Figure 1b. The proportionality of CsCu_2_I_3_ is on par with that of SrI_2_:Eu^2+^, showing a deviation of maximum 4%. Moreover, both are significantly closer to the ideal response in comparison to CsI:Tl^+^.

**Figure 2:**
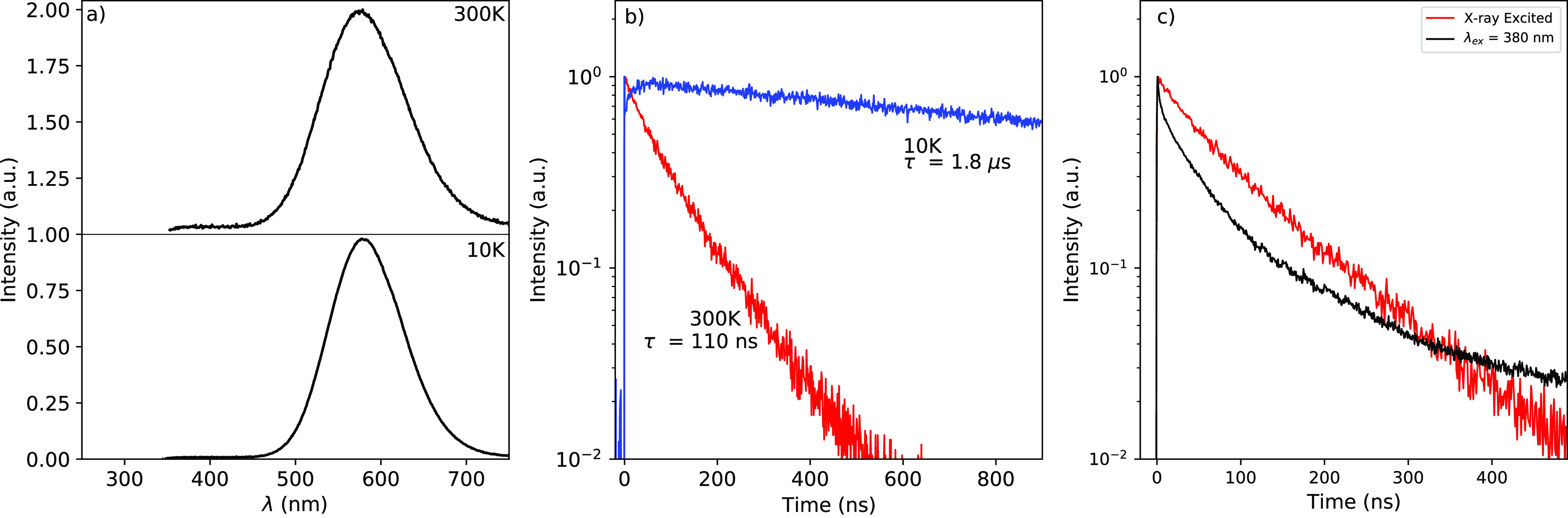
(a) X-ray excited emission spectra of CsCu_2_I_3_ at 300 and 10 K. (b) Pulsed X-ray excited decay curves at 300 and 10 K. (c) Pulsed X-ray excited decay curve at 300 K compared to a decay curve excited at 380 nm at 300 K.

The 300 and 10 K X-ray excited emission spectra are shown in Figure 2a. At 300 K, one broad emission peak is observed located at 570 nm. This agrees with the 300 K X-ray excited emission spectrum presented by Cheng et al.^40^ The emission peak shifts to 575 nm at 10 K. The 570 nm emission falls within the wavelength range where the detection efficiency of the APD is at its maximum. Thus, as described by Wolszczak et al., the number of detected photons from a scintillation event is increased compared to the detection with a PMT.^17^

Figure 2b shows the 300 and 10 K decay curves under pulsed X-ray excitation. At both temperatures, the decay curves show single-exponential behavior. At 300 K, the lifetime is 110 ns, increasing to 1.8 μs at 10 K. The 300 K lifetime, under pulsed X-ray excitation, is approximately 50 ns slower compared to reported lifetimes under UV–vis excitation.^34,35,37,38^ A comparison between the 300 K decay curve measured under pulsed X-ray excitation and excitation by a 380 nm pulsed laser, detecting all photons with a wavelength of >425 nm, is shown in Figure 2c. The optically excited decay curve shows a similar nonexponential shape compared to the reported decay curves for CsCu_2_I_3_ single crystals.^40,43,47^

**Figure 3:**
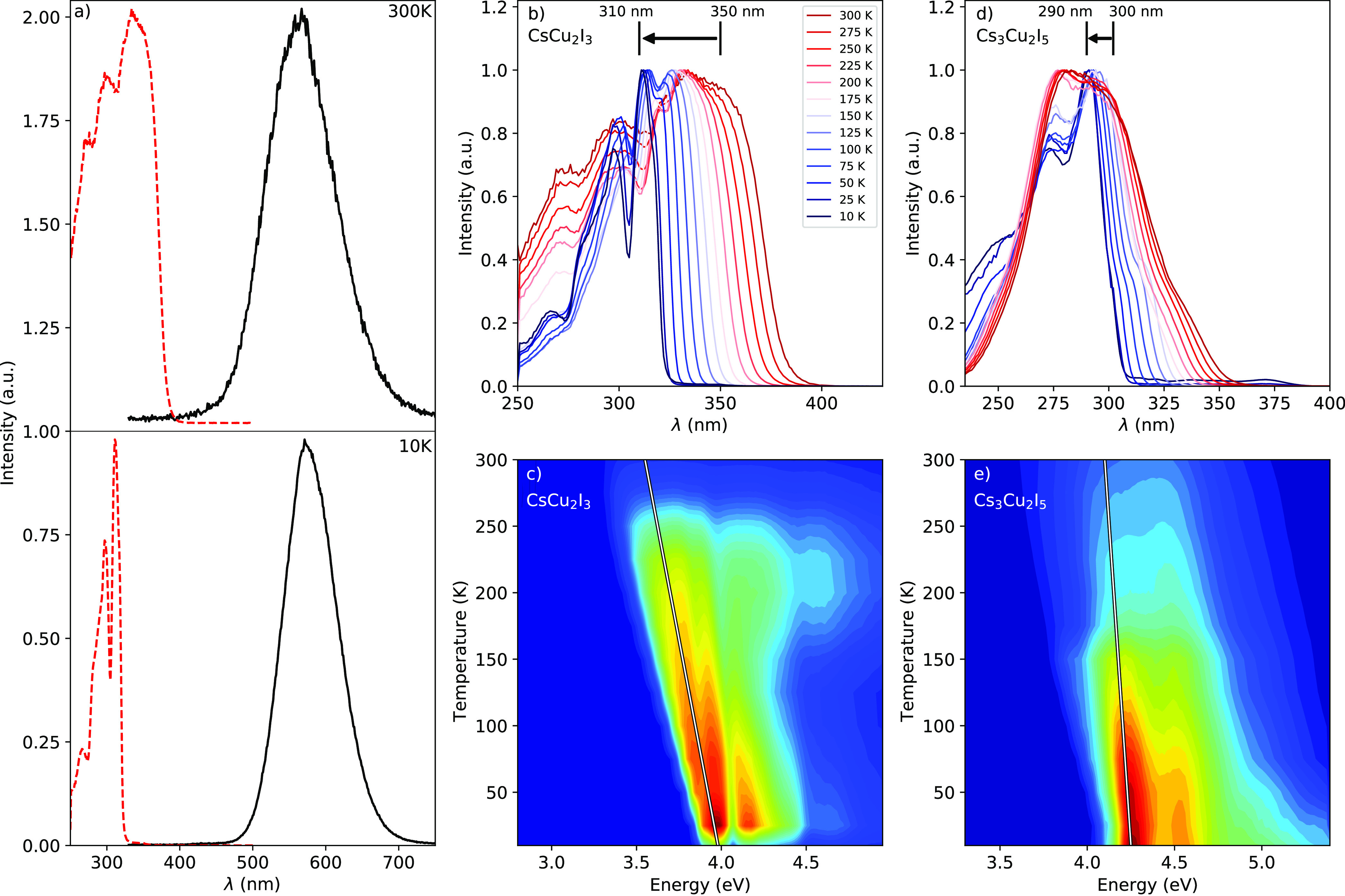
(a) Photoluminescence emission (black line, λ_ex_ = 300 nm) and excitation (red dashed line, λ_em_ = 577 nm) spectra of CsCu_2_I_3_ at 300 and 10 K. (b) Temperature-dependent photoluminecence excitation spectra of CsCu_2_I_3_ (λ_em_ = 577 nm) from 10 to 300 K. (c) Temperature-dependent photoluminecence excitation spectra of CsCu_2_I_3_ on an energy scale. The 2D plot shows the luminescence intensity on a linear scale from blue (low) to red (high). The white line indicates the shift of the lowest energy peak in the excitation spectra in (c) and (e). (d) Temperature-dependent photoluminecent excitation spectra of Cs_3_Cu_2_I_5_ (λ_em_ = 445 nm) from 10 to 300 K. The same color annotation applies to (b) and (d). (e) Temperature-dependent photoluminecence excitation spectra of Cs_3_Cu_2_I_5_ on an energy scale. The 2D plot has the same intensity scaling as in (c).

The 300 and 10 K photoluminescence emission (PL) and photoluminescence excitation (PLE) spectra are shown in Figure 3a. At 300 K, one broad emission peak is observed at 560 nm, shifting to 570 nm at 10 K. The 300 K excitation spectrum shows four peaks located at 265, 300, 330, and 350 nm. The 330 and 350 nm peaks merge and shift to 310 nm at 10 K, while the other peaks show no shift. From Figure 3a it can be observed that CsCu_2_I_3_ has a large Stokes shift of 1.49 eV at 300 K, therefore preventing self-absorption-related losses. At 10 K, the Stokes shift increases to 1.82 eV. These features, the large Stokes shift and broad emission bands, are often attributed to self-trapped exciton (STE) emission.^48^ The 300 K excitation and emission spectra are in good agreement with previously reported spectra.^34−36,40,41^

The temperature-dependent change of the Stokes shift is mainly caused by the shift of the fundamental absorption edge. This is clearly visible in Figure 3b and c, showing temperature-dependent PLE spectra of CsCu_2_I_3_ from 300 to 10 K. Upon cooling, the fundamental absorption edge starts to shift toward shorter wavelengths. The way in which this happens, however, is significantly different compared to the shift of the fundamental absorption edge observed in the temperature-dependent PLE spectra measured for Cs_3_Cu_2_I_5_. The latter is shown in Figure 3d and e.

**Figure 4:**
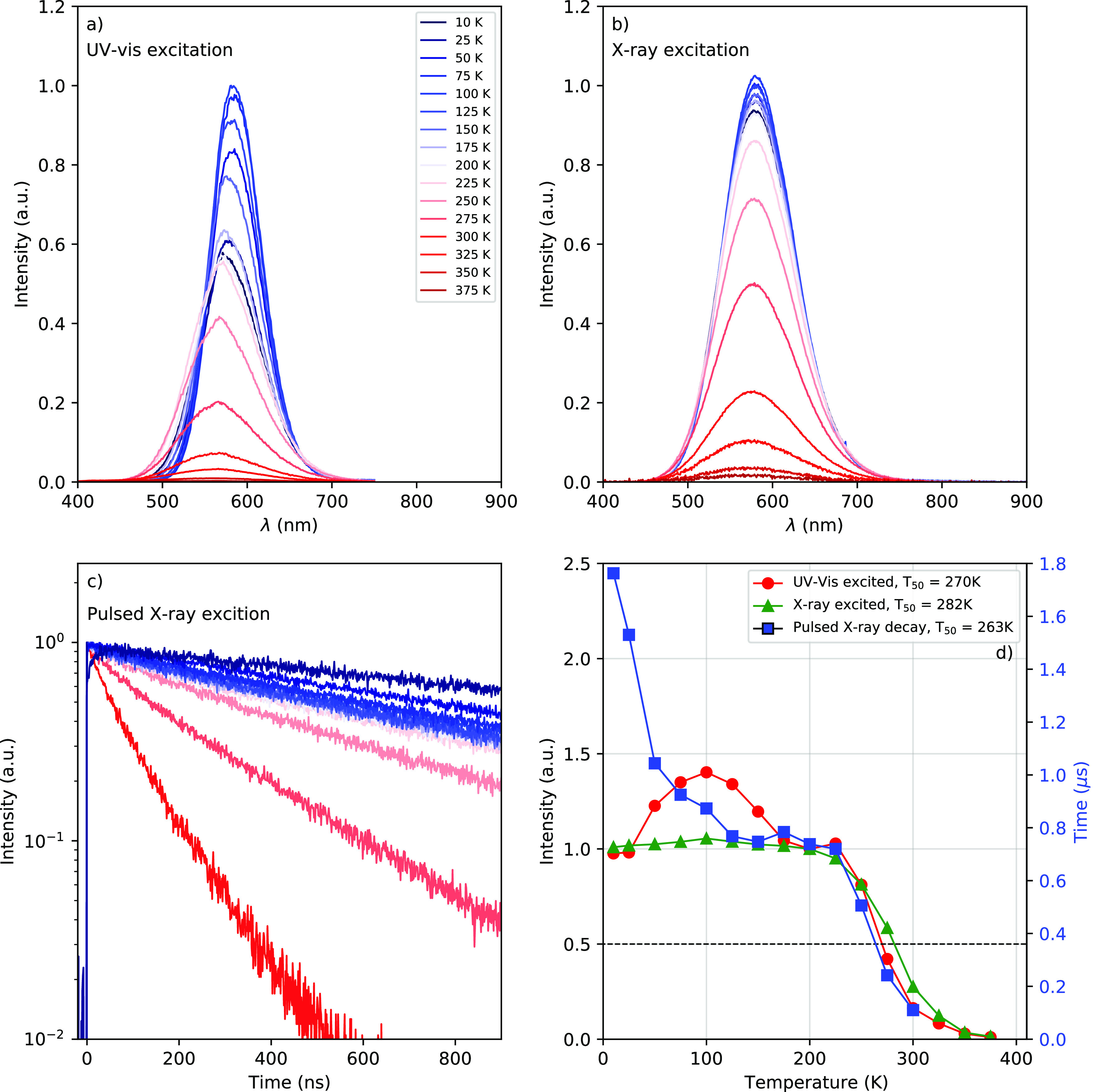
(a) Temperature-dependent photoluminescence emission spectra (λ_ex_ = 300 nm) from 10 to 375 K. The temperature legend in (a) also applies to (b) and (c). (b) Temperature-dependent X-ray excited emission spectra from 10 to 375 K. (c) Temperature-dependent pulsed X-ray excited decay curves from 10 to 300 K. (d) Integrated emisison intensity from the temperature-dependent photoluminescence emission (red circles, left axis) and X-ray excited emission (green triangles, left axis) measurements, normalized at 200 K, and life times obtained from the temperature-dependent pulsed X-ray excited decay measurements (blue squares, right axis).

Figure 4a–c shows the temperature-dependent photoluminescence emission, X-ray excited emission, and pulsed X-ray excited decay curves of CsCu_2_I_3_, respectively. The trends in the temperature behavior are summarized in Figure 4d, showing the quenching curves of the integrated spectral intensity and decay time. All measurements show strong thermal quenching above 200 K. The increase and decrease of the photoluminescence intensity below 200 K result from the strong shift of the PLE spectra upon cooling, as demonstrated in Figure 3b. The latter is not observed under X-ray excitation. The pulsed X-ray excited decay curves show an increase of the decay time from 110 to 740 ns upon cooling from 300 to 200 K. The decay time is constant between 200 and 125 K but increases again to more than a microsecond at 100 K and below.

The quenching curves presented in Figure 4d provide the temperatures (*T*_50_) at which the intensity and decay time have dropped to 50% of their low temperature value. *T*_50_ values of 270, 282, and 263 K were determined for the photoluminescence emission, X-ray excited emission, and pulsed X-ray excited decay, respectively. The APD used for the pulse height spectrum, shown in Figure 1a, is cooled to 260 K in order to reduce noise and prevent gain drift. The temperature of the sample is estimated to be close to 260 K. Hence, the pulse height spectra shown in Figure 1a were measured around the *T*_50_ point of the quenching curves. This suggests that the light yield could increase by a factor of 2 by cooling the sample.

**Figure 5:**
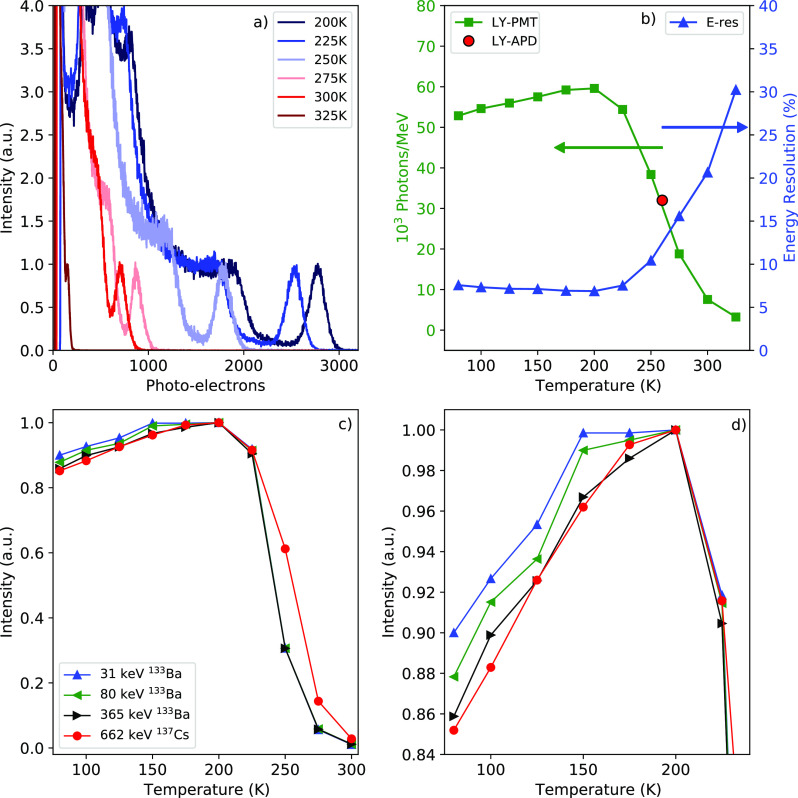
(a) Temperature-dependent pulse height spectra, from 325 to 200 K. (b) Light yield obtained from the temperature-dependent pulse height measurements from 325 to 80 K (green squares, left axis). The red circle indicates the light yield obtained from the pulse height spectrum measured on an APD. Energy resolution obtained from the temperature-dependent pulse height measurements (blue triangles, right axis). (c) Temperature-dependent pulse height spectra, from 300 to 80 K, using 662 keV γ-photons from ^137^Cs and 31, 80, and 365 keV γ-photons from ^133^Ba. All curves are normalized on the light yield at 200 K. (d) Zoom in of the temperature-dependent pulse height spectra in (c) from 80 to 200 K. The legend in (c) also applies to (d).

The effect of temperature on the light yield is studied experimentally via a series of pulse height measurements from 325 to 80 K using 662 keV γ-photons of ^137^Cs. The measurements are performed using a PMT. Figure 5a shows the pulse height spectra from 325 to 200 K. From the latter, it can be observed that the number of detected photoelectrons increases upon cooling, corresponding with an increase of the light yield. The change in light yield and energy resolution between 80 and 325 K is shown in Figure 5b. Between 325 and 200 K the light yield shows quenching behavior similar to the curves presented in Figure 4d, yielding a *T*_50_ of 262 K, which is very close to the values obtained from Figure 4d. The temperature-dependent light yield reaches its maximum of 60 000 photons/MeV at 200 K, corresponding to the detection of 2760 photoelectrons. If we manage to engineer the emission such that T_50_ increases to 350 K, one might increase the light yield toward 60 000 photons/MeV. The light yield obtained from the pulse height spectrum measured on an APD, shown in Figure 1a, falls in line with the curve shown in Figure 5b and is indicated by the red circular marker. From 200 to 80 K, the light yield starts to decrease, going from 60 000 to 52 800 photons/MeV.

Coinciding with the increase of the light yield between 325 and 200 K, the energy resolution improves from 30% to 6.8%, respectively. The measured energy resolutions in this experiment are higher compared to the one shown in Figure 1a. This is the direct result of geometric restrictions imposed by the cryostat. The sample could not be mounted directly on the entrance window of the PMT, combined with the less suitable match of the PMT detector efficiency with the mean emission wavelength of CsCu_2_I_3_.

The pulse height measurements with 662 keV γ-photons, as shown in Figure 5a and b, were extended by measurements with 31, 80, and 365 keV X-ray and γ-photons of ^133^Ba to study the effect of the deposition energy. The resulting curves are shown in Figure 5c and d. Above 200 K, all curves show the same quenching behavior as observed in Figure 4b and 5b. Below 200 K, the light yield decreases, but the reduction is less for smaller deposition energies (see Figure 5d).

## 3 Discussion

The shapes of the room temperature X-ray and photoexcited decay curves are different, as shown in Figure 2c. Under pulsed X-ray excitation of CsCu_2_I_3_, a single exponential decay curve is observed. However, upon excitation with a 380 nm pulsed laser, the decay curve shows nonexponential behavior. This nonexponential behavior is observed for both single crystals and films of CsCu_2_I_3_.^34−38,43,47^ Zhang et al. and Mo et al. suggested that the nonexponential shape stems from excitation of surface trap states and bulk STE emission.^36,43^ Based on the aforementioned and the significantly larger penetration depth of X-rays versus optical photons, it is suggested that the single exponential decay curve observed under pulsed X-ray excitation results solely from bulk STE emission.

Based on the temperature-dependent photoluminescence excitation spectra shown in Figure 3 it was observed that the fundamental absorption edge of CsCu_2_I_3_ blue shifts in a different way compared to Cs_3_Cu_2_I_5_. This difference is further investigated by determining the change in the band gap as a function of temperature based on the shift of the lowest-energy peak in the excitation spectra. The position of these peaks at 300 and 10 K, combined with the direction of the shift, are indicated in Figure 3b and d for CsCu_2_I_3_ and Cs_3_Cu_2_I_5_, respectively. It was determined that the band gap of CsCu_2_I_3_ shifts from 4 eV at 10 K to 3.55 eV at 300 K, corresponding to a change of 155 meV/100 K. The band gap of Cs_3_Cu_2_I_5_ shifts from 4.25 eV at 10 K to 4.1 eV at 300 K, corresponding to a change of 52 meV/100 K. The temperature-dependent band gap change is visualized by a white line plotted in Figure 3c and e. The band gap change of Cs_3_Cu_2_I_5_ is very similar to values reported for more traditional semiconductors (50–100 meV/100 K)^49−51^ like silicon or more modern semiconductors (50 meV/100 K) like lead halide perovskites.^52,53^ The band gap change observed for CsCu_2_I_3_, however, is approximately 2–3× larger compared to these values.

The shifts determined from temperature-dependent excitation spectra of Cs_3_Cu_2_I_5_ show classical behavior, as shown in Figure 3d; the peaks in the spectrum become broader at higher temperature. The behavior of CsCu_2_I_3_ is significantly different. From Figure 3a–c, it can be observed that the 330 and 350 nm peak observed at 300 K starts to blue shift upon cooling and merges with the 310 nm peak that appears below 200 K, resulting in the large bang-gap shift. The valence band maximum is formed by the Cu 3d and I 5p orbitals, and the conduction band minimum is formed by the Cu 4s and I 5p orbitals for both CsCu_2_I_3_ and Cs_3_Cu_2_I_5_.^36,37,39^ The main difference between these compounds lies in their crystallographic structure and the related electronic structure: Cs_3_Cu_2_I_5_ has a 0D structure with isolated [Cu_2_I_5_]^3–^ units built from two face-sharing tetrahedra, whereas CsCu_2_I_3_ has a 1D structure with double chains [Cu_2_I_3_]^−^ of edge-sharing tetrahedra.^39^ The fundamental origin for the large band gap change of CsCu_2_I_3_ remains unclear.

The temperature-dependent light yield measurements presented in Figure 5 b show an increase of the light yield from 325 to 200 K, reaching 60 000 photons/MeV. From 200 to 80 K the light yield decreases by 12% to 52 000 photons/MeV. Coinciding with this decrease, the energy resolution deteriorates from 6.8% at 200 K to 7.6% at 80 K. Between 200 and 80 K, the band gap changes by 0.19 eV, from 3.7 eV at 200 K to 3.89 eV at 80 K.

The theoretical light yield of a material, as shown in eq 1, depends on its band gap energy.^1,54^ Here *N*_eh_ is the number of created electron hole pairs in the scintillator per MeV deposited ionization energy, β is usually taken to be ≈2.5, and *E*_g_ corresponds to the band gap energy. Based on eq 1 and the observed increase of the band gap, it is estimated that the theoretical light yield decreases by 5% from 200 to 80 K. This only partially explains the observed 12% decrease of the light yield in Figure 5b.
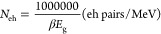
1

The decrease in the light yield below 200 K is not observed in the X-ray excited emission spectra shown in Figure 4d. These spectra are recorded using continuous X-ray excitation with an average energy of 40 keV. This difference could be explained either due to the different excitation energies, the different integration times used, or the different experimental setups.

The influence of the deposition energy is studied by recording temperature-dependent pulse height spectra using 662 keV γ-photons from ^137^Cs and 31, 80, and 365 keV γ-photons from ^133^Ba, keeping the sample in the same position for all measurements. The resulting light yields as a function of temperature and deposition energy are shown in Figure 5c and d. Above 200 K, all curves show the same quenching behavior as that observed in Figure 4b. Below 200 K all curves show a decrease of the light yield. However, the magnitude of this decrease depends on the deposition energy, as shown in Figure 5d. The light yield decreases by approximately 15% between 200 and 80 K upon excitation with 662 keV γ-photons but only by 10% upon excitation with 31 keV γ-photons. Nonetheless, this change is not the same as that observed in Figure 4d under continuous X-ray excitation.

## 4 Conclusion

In this work, the emerging scintillator CsCu_2_I_3_ has been characterized as a function of temperature. Using an APD, to match the detection efficiency to the mean emission wavelength of CsCu_2_I_3_, an energy resolution of 4.8% and a light yield of 36 000 photons/MeV have been measured for 662 keV excitation. Using different deposition energies, it is demonstrated that the nonproportionality of CsCu_2_I_3_ is on par with that of SrI_2_:Eu^2+^. At 300 K, CsCu_2_I_3_ has a Stokes shift of 1.49 eV and shows only one emisison peak centered around 560 nm. This mean emission wavelength fits well with the spectral sensitivity of modern Si-based photodetectors with higher detection efficiencies compared to more traditional PMTs. At 300 K, a lifetime of 110 ns has been measured under pulsed X-ray excitation.

From temperature-dependent photoluminescence emission, X-ray excited emission, and pulsed X-ray excited decay measurements, *T*_50_ values of 270, 282, and 263 K have been determined, respectively. Accordingly, the CsCu_2_I_3_ emission is already significantly quenched at 300 K. Using temperature-dependent pulse height measurements, it was proofed experimentally that the light yield of CsCu_2_I_3_ increases to 60 000 photons/MeV at 200 K. Below 200 K, the light yield decreases again by 10% to 15% down to 80 K, depending on the deposition energy. The decrease in the light yield below 200 K is attributed to the change in the band gap energy by 155 meV/100 K. The exact nature of this large change could not be explained. Engineering CsCu_2_I_3_ by chemical variation and optimization of the crystal growth process might shift the *T*_50_ above 300 K and improve the room temperature scintillation properties of CsCu_2_I_3_.

## 5 Experimental Section

Crystals of CsCu_2_I_3_ and Cs_3_Cu_2_I_5_ were grown from stoichiometric mixtures of CsI and CuI using the vertical Bridgman technique with a static ampule and a moving furnace. CsI (Merck 99.5%) and CuI (ABCR, 99.999%) were dried in vacuum at 200 °C. Stoichiometric amounts of the iodides, about 5 g per sample, were sealed in silica ampules under vacuum. The ampules were heated to 10 K above the melting point of the ternary compound, and the temperature was kept for 1 day. The crystal growth was started by slowly moving the furnace up by about 15 mm/day. The ampule cooled to room temperature within 10 days. CsCu_2_I_3_ melts congruently at 383 °C.^44^ The melting point of Cs_3_Cu_2_I_5_ is at about 390 °C.^44^ All handling of starting materials and products was done in glove boxes with H_2_O and O_2_ below 0.1 ppm. For spectroscopic measurements, crystals were sealed in silica ampules under He or in sample containers under inert gas or vacuum. The crystal structure and the phase purity of the samples were confirmed by powder XRD.

Pulse height spectrum and nonproportionality curves were recorded using an Advanced Photonix APD (type 630-70-72-510) operating at a bias voltage of 1560 V. The APD was stabilized at 260 K to prevent gain drift. The signal was fed to a Cremit CR-112 preamplifier before being processed by an Ortec 672 spectroscopic amplifier, with a shaping time of 3 μs, and digitized by an Ortec AD144 26K ADC. The light yield was calculated based on the channel position of the photopeak maximum and that of the peak from direct detection of 17.8 keV X-rays of ^241^Am, as described by dDe Haas and Dorenbos.^45^

X-ray emission spectra were recorded using a tungsten anode X-ray tube operating at 79 kV, producing X-rays with an average energy of 40 keV. The low energy side of the X-ray spectrum was filtered out by a 3 mm aluminum filter to prevent radiation damage in the sample. The samples were mounted on the coldfinger of a closed cycle helium cryostat.

Pulsed X-ray excited decay curves were measured via a time-correlated single photon counting method. The start signal was generated by a PicoQuant LDH–P-C440 M pulsed laser, directly hitting a Hamamatsu N5084 light-excited X-ray tube to create X-ray pulses with an average energy of 18.2 keV. The stop signal was generated upon detection of a single photon by using an ID Quantique id100–50 single-photon counter. The start and stop signals were processed by an Ortec 567 time-to-amplitude converter, whose output was digitized by an Ortec AD 144 16K ADC. The samples were mounted on the coldfinger of a closed cycle helium cryostat.

Time resolved photoluminescence spectra were measured via the time-correlated single photon counting method. A PicoQuant LDH-P-C-375 M pulsed diode laser was used to excite the sample. The reference output of the PicoQuant laser driver was used as the start signal and was fed to an Ortec 567 time-to-amplitude converter. The emitted light was detected by an ID Quantique id100-50 single-photon counter. The final signal was digitized by an Ortec AD144 amplitude to digital converter.

Photoluminescence emission and excitation spectra were recorded using a 450 W xenon lamp and Horiba Gemini 180 monochromator to excite the sample. The emitted light was collected at a 90° angle with respect to the excitation source. Reflected excitation light was removed with an optical filter. The emission light passed through a Princeton Instruments SpectraPro-SP2358 monochromator connected to a Hamamatsu R7600U-20 PMT. All spectra were corrected for the lamp intensity. The samples were mounted on the coldfinger of a closed cycle helium cryostat.

Temperature-dependent pulse height spectra were recorded by mounting the sample on a parabolic stainless steel reflector covered with aluminum foil to increase the reflectivity. The reflector was mounted on a Janis VPF-700 cryostat. The sample chamber was kept under vacuum below 10^–5^ mbar. A Hamamatsu Super Bialkali R6231–100 (SN ZE4500) PMT was used to detect the scintillation photons. It was placed close to the window on the outside of the sample chamber. The distance between the sample and PMT was approximately 5 cm. The light yield was determined based on a comparison with a (Lu,Y)_2_SiO_5_:Ce^3+^ reference sample measured inside the cryostat under identical experimental conditions. The light yield of (Lu,Y)_2_SiO_5_:Ce^3+^ was determined by PMT read out based on the methode described by De Haas and Dorenbos.^45^ The light yield is corrected for the differences in emission wavelength between (Lu,Y)_2_SiO_5_:Ce^3+^ and CsCu_2_I_3_ and the PMT detection efficiency.
